# Transcranial electrical stimulation modulates emotional experience and metabolites in the prefrontal cortex in a donation task

**DOI:** 10.1038/s41598-024-64876-x

**Published:** 2024-06-20

**Authors:** Luiza Mugnol-Ugarte, Tiago Bortolini, Bo Yao, Mark Mikkelsen, Marina Carneiro Monteiro, Ana Carolina Andorinho de Freitas Ferreira, Ivanei Bramatti, Bruno Melo, Sebastian Hoefle, Fernanda Meireles, Jorge Moll, Gorana Pobric

**Affiliations:** 1 Cognitive Neuroscience and Neuroinformatics Unit, The D’Or Institute for Research and Education (IDOR), Rio de Janeiro, Brazil; 2 Department of Psychology, Lancaster University, Lancaster, United Kingdom; 3 Department of Radiology, Weill Cornell Medicine, New York, United States of America; 4 Division of Psychology, Communication and Human Neuroscience, The University of Manchester, Manchester, United Kingdom

**Keywords:** Cognitive neuroscience, Social behaviour, Psychology, Human behaviour, Prefrontal cortex

## Abstract

Understanding the neural, metabolic, and psychological mechanisms underlying human altruism and decision-making is a complex and important topic both for science and society. Here, we investigated whether transcranial Direct Current Stimulation (tDCS) applied to two prefrontal cortex regions, the ventromedial prefrontal cortex (vmPFC, anode) and the right dorsolateral prefrontal cortex (DLPFC, cathode) can induce changes in self-reported emotions and to modulate local metabolite concentrations. We employed in vivo quantitative MR Spectroscopy in healthy adult participants and quantified changes in GABA and Glx (glutamate + glutamine) before and after five sessions of tDCS delivered at 2 mA for 20 min (active group) and 1 min (sham group) while participants were engaged in a charitable donation task. In the active group, we observed increased levels of GABA in vmPFC. Glx levels decreased in both prefrontal regions and self-reported happiness increased significantly over time in the active group. Self-reported guiltiness in both active and sham groups tended to decrease. The results indicate that self-reported happiness can be modulated, possibly due to changes in Glx concentrations following repeated stimulation. Therefore, local changes may induce remote changes in the reward network through interactions with other metabolites, previously thought to be unreachable with noninvasive stimulation techniques.

## Introduction

Every day we encounter complex social environments. Some social contexts are perceived as positive and rewarding, while others induce negative feelings such as guilt and regret^1^. Reaction to these contexts can induce a range of actions, from prosocial to avoidance to punishing behaviors^2^. One example of prosocial conduct is human cooperation, which has been the focus of behavioral economics, social psychology and, more recently, neuroscience^3–6^. Pioneering studies of the neural underpinnings of human cooperation have used economic games to establish a basis for investigating human altruistic behaviors^6–10^. Strictly defined, altruistic actions are those voluntarily performed by an agent to benefit another (non-kin) individual, incurring a cost to the altruistic agent^11,12^. From an economic perspective these acts can be defined as costly actions leading to financial gains for another individual^3^. Examples of altruistic behaviors depend on the context, and can include blood donations^13^, effort/time spent to help others^14^ or money donations^15^.

Indeed, humans often sacrifice material benefits to support social causes^16^, and charitable donations can be used as a proxy for altruistic behavior^15^. They can induce the satisfaction derived from voluntary donations^17^. The feelings resulting from altruistic behavior have been related to prefrontal areas^18–20^ and reward and social affiliation circuitry^6,7^. The reward network comprises dorsomedial and dorsolateral prefrontal cortex (DMPFC and DLPFC), medial frontopolar cortex (MFPC), anterior and subgenual cingulate cortices (ACC and SCC), medial and lateral orbitofrontal cortices (MOFC and LOFC) and medial temporal cortices^7,21^; and the subcortical areas including the striatum, hypothalamus, amygdala, lateral habenula, and pallidum^22^.

Furthermore, vmPFC (which includes parts of the medial, frontopolar and subgenual PFC) has often been identified as an area of the brain that is involved in the representation of the value of a stimulus^23,24^. Additionally, altruistic decisions recruit subcortical areas implicated in general reward responses, such as the ventral tegmental area (VTA) and the ventral striatum^7,25^. That is, the altruistic reward network comprises areas of the prefrontal cortex and subcortical areas, because reward and self-other understanding could motivate altruistic decisions^26^.

In contrast to prosocial behaviors, failing to help someone or a worthy cause, can lead to feelings of guilt, which have been shown to engage sectors of the vmPFC such as the SCC and MFPC^27^. The vmPFC receives direct cortical connections from DLPFC. Both vmPFC and DLPFC are connected with subcortical regions involved in emotional responses^28^. Guilt is a powerful emotion that can promote social reparation and prevent socially harmful actions^5^.

While these fronto-mesolimbic networks play a critical role in prosocial behaviors, the interaction of the specific neurotransmitters that mediate these functions is still not well understood^29,30^. Animal studies show a critical role of dopamine (DA) in prosocial behaviors^1^. Moreover, gamma-aminobutyric acid^31^ (GABA) concentrations in the prefrontal cortex have an important role in modulating activity and DA release in the midbrain and striatum^32^.

Non-invasive brain stimulation techniques such as transcranial Direct Current Stimulation (tDCS)^33^ are increasingly used to study the involvement of brain areas in behavioral tasks. tDCS modulates cortical excitability in the underlying cortex by "up-regulating" or "down-regulating" a region of interest^34^. In the motor cortex, tDCS application of 1 mA, generally, results in depolarisation of the neurons underneath the anode, hence causing an excitatory effect^35^. In contrast, tDCS causes hyperpolarization underneath the cathode and thus inhibition of cortical neurons in the motor cortex^36^. However, the role of cathodal inhibition effects has been debated when applied over different brain areas and/or different stimulation intensities^37,38^^.^ Applying tDCS at a current strength of 2 mA causes excitability increases under both anode and cathode in the motor cortex^39^. It is less clear whether other cortical regions such as the prefrontal cortex also show the reversal of inhibitory effects following 2 mA stimulation.

Behaviorally, 1 mA tDCS protocols, have been shown to modulate emotional pain^40^ negative emotion perception, and to boost emotion regulation^41^. Importantly, tDCS applied to the Medial Prefrontal Cortex (MPFC) was demonstrated to influence feelings of guilt and the willingness to perpetrate social violations^38^. Studies applying 2 mA currents in online protocols report facilitation of motor learning and skill acquisition^42–44^. Yet, the behavioral effects of 2 mA tDCS modulation have not been studied consistently in higher cognitive processes.

Magnetic resonance spectroscopy (MRS) can be combined with tDCS to study changes in metabolite concentrations following stimulation. The major excitatory and inhibitory neurotransmitters glutamate (Glu) and GABA have been reported to be involved in secondary tDCS effects in the motor cortex^35,36^. For instance, GABA is involved in anodal tDCS after-effects, while both GABA and Glu concentrations have been modulated following cathodal stimulation^35^. Application of anodal tDCS at 1 mA over the motor cortex in a single-session online paradigm decreases GABA concentrations^35,45,46^, while online 1 mA application over bilateral DLPFC increases Glu, however, these results were short-lived and no changes in metabolite concentrations were seen off-line^47^. Repeated administration of anodal tDCS at 2 mA has decreased Glx (Glu + glutamine [Gln]) levels in DLPFC^48^. Conversely, Alvarez-Alvarado et al. (2021) reported a sustained increase in Glx concentrations after 2 mA tDCS stimulation over bilateral DLPFC during two weeks of working memory training^43^. These somewhat contradictory effects of 2 mA tDCS on higher cognitive processes warrant further exploration. In particular, the effects of tDCS on metabolites have not been explored comprehensively in emotional processing tasks.

**Figure 1:**
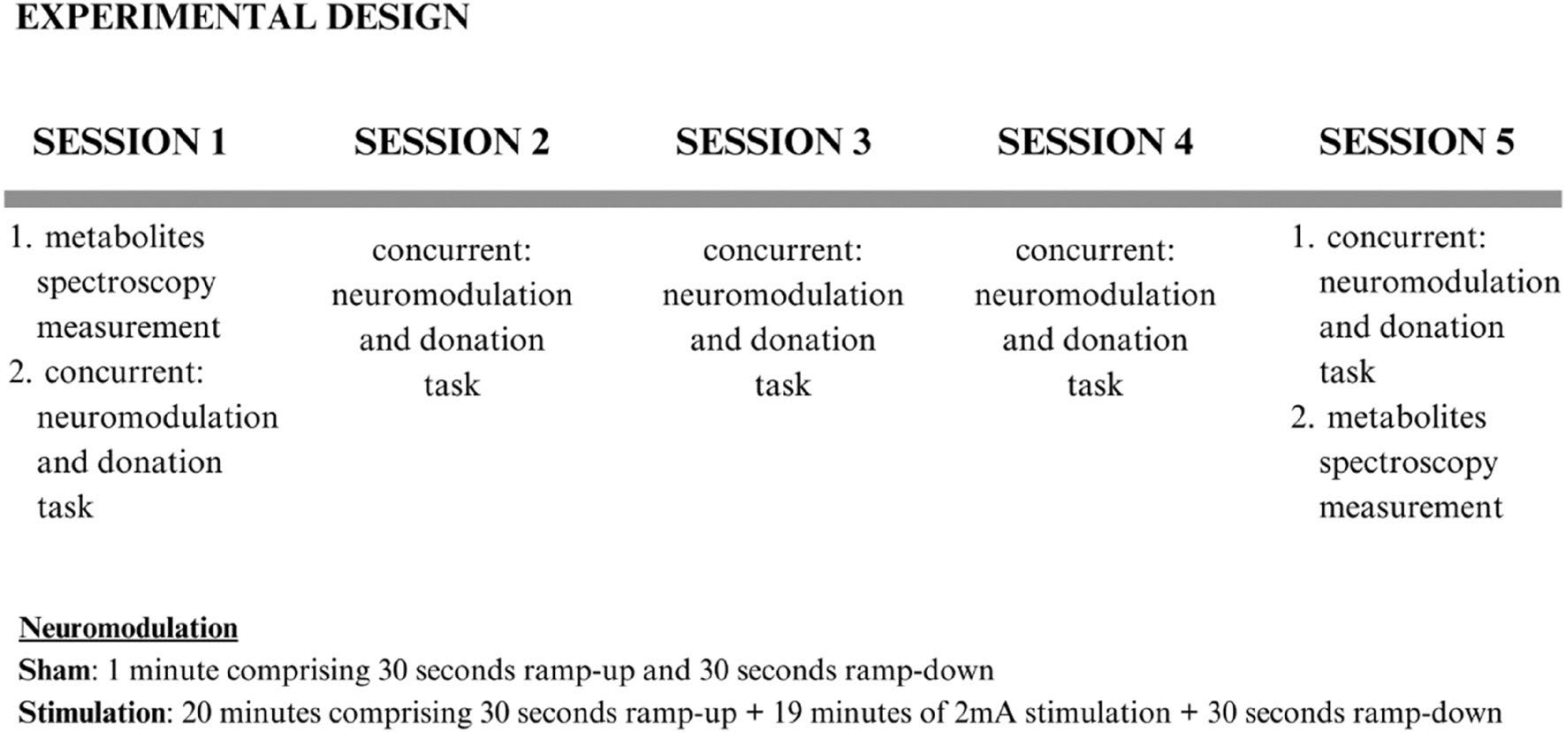
The spectroscopy measurements were acquired before the first stimulation on day 1 (session 1) and after the last stimulation on day 5 (session 5). Following the first spectroscopy acquisition on session 1, participants performed the donation task concurrently with the stimulation. On sessions 2–4, participants were engaged in a donation task concurrently with the stimulation. On session 5, participants performed a donation task concurrently with the stimulation, and were subsequently taken to the MRI, where post-stimulation spectroscopy measurement was obtained. Neuromodulation: Sham group: 1 min comprising 30 s ramp-up and 30 s ramp-down; Stimulation group: 20 min comprising 30 s ramp-up + 19 min of 2 mA stimulation + 30 s ramp-down.

In this study, we used tDCS in combination with MRS to explore the neural mechanisms of two emotions associated with altruistic donations, by modulating neural activity in vmPFC and DLPFC. We aimed to replicate the findings of Chib et al.^49^, who hypothesized that their tDCS protocol stimulated ventral midbrain areas which were of interest to our study. We used their protocol parameters (e.g. 2 mA current intensity) and a specific tDCS electrode montage where both electrodes were stimulating^49^ concurrently vmPFC and DLPFC (Fig. 1). tDCS was applied from the start of the session for 20 min while participants were engaged in a modified Dictator Game (adapted from ^7^), in which participants decided how much money they would like to donate to different Non-Governmental Organizations (NGOs). We tested whether tDCS affects happiness and guiltiness in our participants in the context of costly altruistic decisions. Stimulation was expected to increase perceived happiness while diminishing perceived guilt. Importantly, MRS was used to estimate the effect of 2 mA stimulation on metabolite concentration in the PFC under both anode and cathode. We expected that, over five sessions of non-invasive brain stimulation, changes would be observed in GABA and Glx concentrations in vmPFC and DLPFC. In line with^50^ we expected an increase of GABA following 2 mA tDCS stimulation overof Glx in DLPFC^48^.

## Results

Each MRS dataset was visually inspected for data quality and signal artifacts. Following our data exclusion criteria^51^, a total of 17 participants (11 cisgender women; 8 from the active group) were excluded (14 from the vmPFC dataset; 3 from the DLPFC dataset) based on poor single-to-noise ratio (SNR) of the 3 ppm GABA + peak or high model fit error (> 20%). Before analyses, participants were excluded for each stimulation site if their GABA and/or Glx measures were more than 2 standard deviations away from the mean for each session (baseline, post-stimulation) and stimulation group (active, sham). These procedures resulted in the loss of a further 6 participants in the vmPFC dataset (final N = 20, with 10 in the active group and 10 in the sham group) and a further loss of 3 participants in the DLPFC dataset (final N = 34, with 17 in the active group and 17 in the sham group). This means, all the analyses—MRS and behavioral—were run only for the participants with good quality spectroscopy data. The higher proportion of excluded participants based on the vmPFC signal is expected, given the higher susceptibility effects in this region, which affects SNR. The analysis consisted of four steps: 1) GannetLoad, in which the data were loaded and processed; 2) GannetFit, in which the area under the edited GABA signal at 3 ppm and Cr signal at 3 ppm were estimated (see Methods Fig. 8); and 3) GannetCoRegister, in which the MRS voxels were co-registered with the *T*_1_-weighted structural image.

### *Behavioral analyses: the effects of Session and Stimulation on reported happiness and guiltiness (N* = *40)*

To assess the effects of session and stimulation on money donations, and reported happiness and guiltiness associated with donated money, we fitted linear mixed-effects models (LMEMs) for the mean amount of donation over 5 days, and each respective emotion using the lmer() function of the lme4 package^52^. All models shared the same fixed-effect and random-effect structures. The former included the fixed factors of Session (Day 1, 2, 3, 4, 5) and Group (active, sham) and their interactions; both factors were deviation coded, with a mean of 0 and a SD of 0.5. The latter employed the maximal random-effect structure by design, including a by-Subject random intercept and a by-Subject random slope for Session. The *p*-values for the fixed effects were computed using Satterthwaites’s approximation using the lmerTest package^53^.

A linear mixed model with average donation as the dependent variable and the factors session, group, and their interaction as fixed effects and participant and session as random effects did not find any significant results: For money donations, we did not find any significant results of Session (b = 0.08929, SE = 0.93543, t = 0.095, *p* = 0.924); Group (b = 2.57793, SE = 2.83140, t = 0.910, *p* = 0.368), nor a Session x Group interaction (b = 0.05170, SE = 1.87568, t = 0.028, *p* = 0.978.).

**Figure 2:**
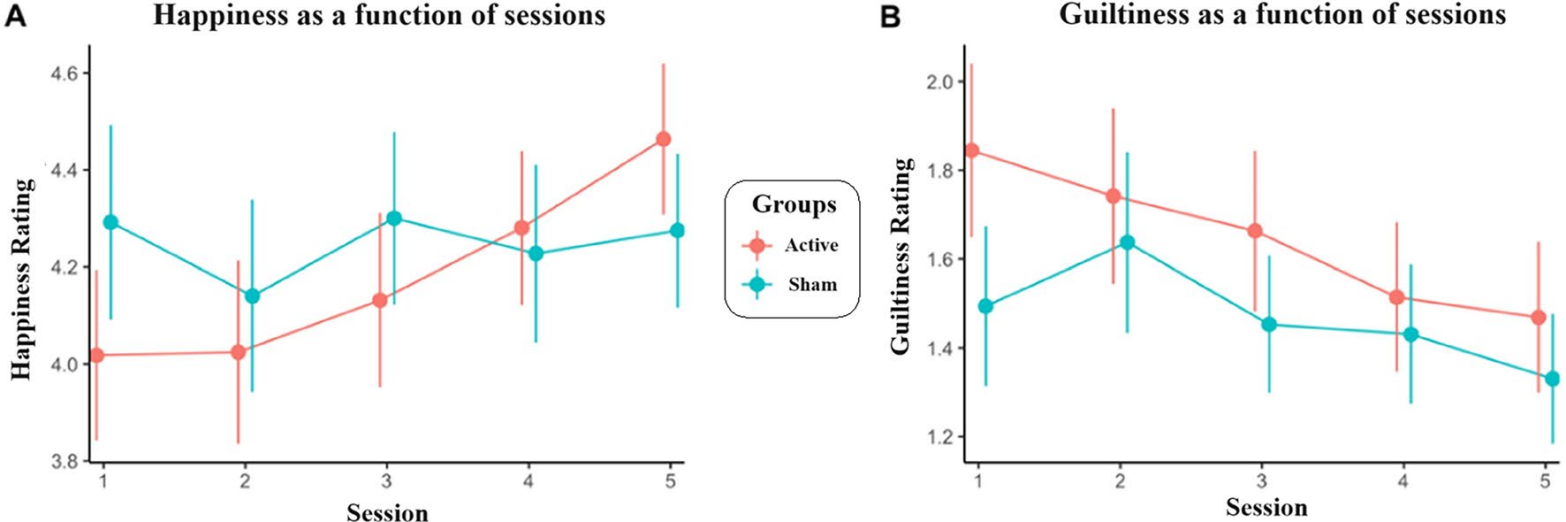
Mean happiness (**A**) and guiltiness (**B**) ratings over time and between groups; error bars indicate standard errors.

For reported happiness, there was a significant main effect of Session (*b* = 0.178, *SE* = 0.064 *t* = 2.773, *p* = 0.009), and a significant Session × Group interaction (*b* = 0.311, *SE* = 0.129, *t* = 2.417, *p* = 0.021). Specifically, reported happiness increased significantly over time, however only in the active group (*b* = 0.325, *95%CI* = [0.146 0.504]) and not in the sham group (*b* = 0.015, *95%CI* = [-0.173 0.203]). The main effect of Group was not significant (*p* = 0.712), suggesting that happiness did not significantly differ between groups overall which may be explained by the lower starting happiness ratings in the active group (Fig. 2A).

For reported guiltiness, there was a main effect of Session (*b* = -0.218, *SE* = 0.080, *t* = − 0.2.735, *p* = 0.009). The main effect of Group and the Session × Group interaction were not significant, *p*s > 0.287. These findings suggest that reported guiltiness decreased significantly over time, irrespective of stimulation (Fig. 2B).

### *MRS analyses: the effects of session and stimulation on GABA and Glx levels in vmPFC (N* = *20) and DLPFC (N* = *34).*

We fitted four LMEMs (GABA-vmPFC, GABA-DLPFC, Glx-vmPFC, Glx-DLPFC) using the lmer() function of the lme4 package^52^. All models shared the same fixed-effect and random-effect structures. The fixed-effect structure included fixed factors of Session (post-intervention, baseline) and Group (active, sham) as well as their interactions; all factors were deviation coded, with a mean of 0 and a SD of 0.5. The random-effect structure included a by-Subject random intercept. The *p*-values for fixed effects were computed using Satterthwaites’s approximation using the lmerTest package^53^.

**Figure 3:**
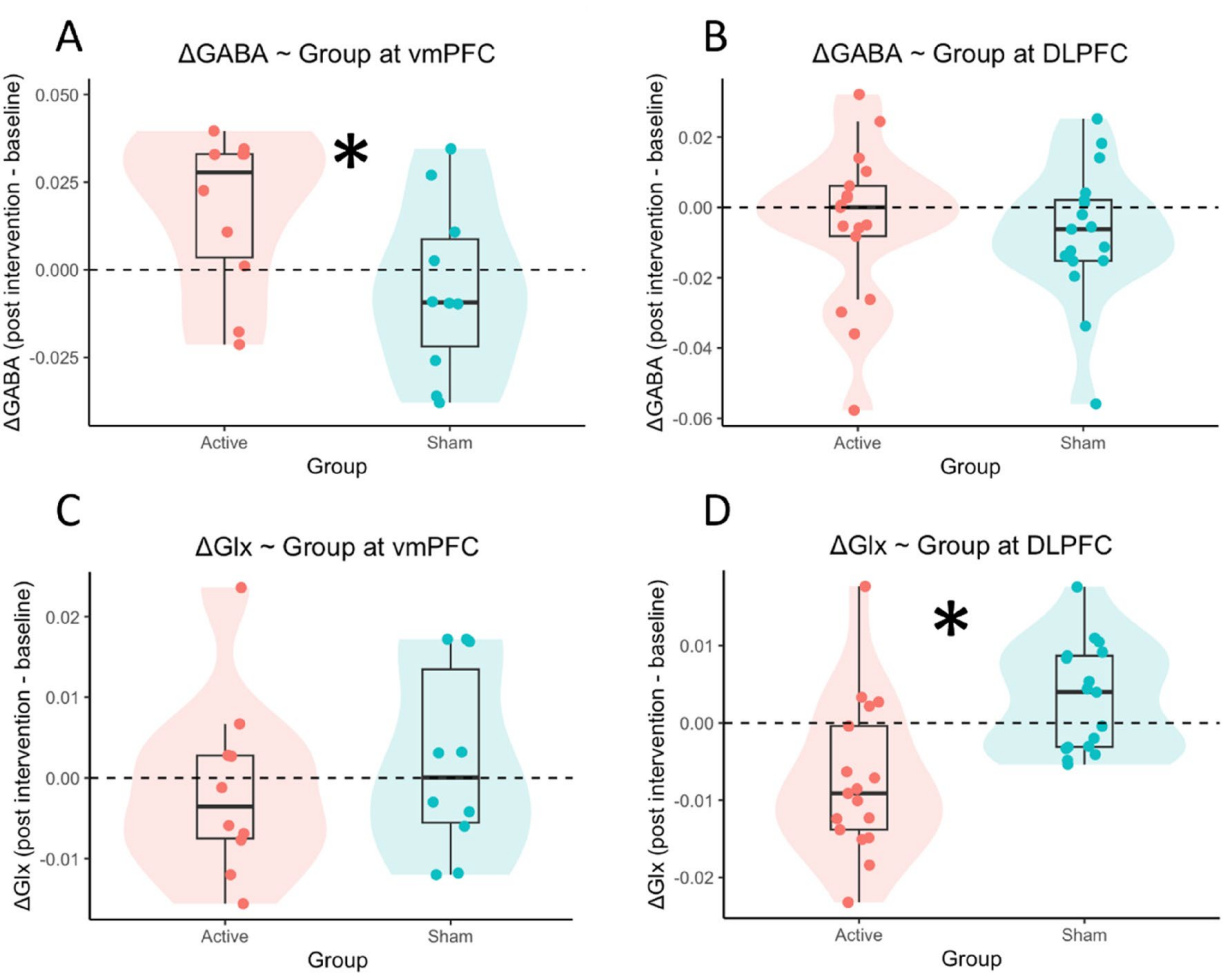
Changes in GABA (upper row; labelled Δ_GABA) and Glx (lower row; labelled Δ_Glx) post-intervention (session 5—session 1) at vmPFC (left column) and at DLPFC (right column). Active group in red, sham group in green. Jittered dots represent individual observations in each group, with their distributions illustrated by violin plots. Boxplots indicate the median and the 1st and 3rd quartiles of the distributions.

For GABA at vmPFC (Fig. 3A), there was a significant two-way interaction between Session and Group (*b* = 0.022, *SE* = 0.010, *t* = 2.152, *p* = 0.038). GABA levels significantly increased post intervention in the active group, ΔGABA = 0.017, *95%CI* = [0.002 0.032], but did not significantly change in the sham group, ΔGABA = − 0.005, *95%CI* = [− 0.021 0.010]. No other effects were significant, *p*s > 0.271.

For GABA at DLPFC (Fig. 3B), there was a non-significant main effect of Session (*b* = − 0.006, *SE* = 0.004, *t* = − 1.704, *p* = 0.098), indicating a trend that GABA levels decreased post intervention, irrespective of Group. No other effects were significant, *p*s > 0.541.

For Glx at vmPFC (Fig. 3C), there was a marginal main effect of Group (*b* = − 0.007, *SE* = 0.004, *t* = − 2.046, *p* = 0.056), indicating a trend that the overall Glx levels were lower in the active group than in the sham group. No other effects were significant, *p*s > 0.511.

For Glx at DLPFC (Fig. 3D), there was a significant Session × Group interaction (*b* = − 0.011, *SE* = 0.003, *t* = − 3.578, *p* = 0.001). Glx significantly decreased post intervention in the active group, ΔGlx = − 0.007, *95%CI* = [− 0.012 − 0.003], and did not significantly change in the sham group, ΔGlx = 0.003, *95%CI* = [− 0.001 0.007]. There was also a marginal main effect of group (*b* = 0.004, *SE* = 0.002, *t* = 1.962, *p* = 0.058), indicating that the overall Glx levels were higher in the active group than in the sham group. The main effect of Session was not significant, *p* = 0.156.

We also examined the potential relationship between prefrontal metabolic changes due to the stimulation protocol and the emotions following altruistic experience. The results can be found in (Supplementary Material 2).

## Discussion

In this study we used tDCS over both vmPFC and DLPFC to examine self-reported feelings of happiness and guiltiness, as well as changes in GABA and Glx, after participants were engaged in a donation task for 5 consecutive days. We have shown that simultaneous tDCS anodal stimulation at 2 mA over vmPFC and cathodal stimulation over DLPFC influenced the self-reported happiness following a donation-task. There was an increase in happiness in the active group and a decrease in the feeling of guilt in both groups across sessions. Importantly, we report increased levels of GABA in the vmPFC of the active group as a result of 2 mA tDCS stimulation over five sessions.

The Glx level decreased in the vmPFC of the active group. The observation that repeated tDCS causes a decrease of excitatory neurotransmitters implies a difference in the underlying mechanisms between repeated tDCS and single stimulation, in line with^48^. Furthermore, cathodal stimulation of DLPFC might have suppressed its control over vmPFC, which resulted in an enhancement of the anodal effect in the latter. This enhanced effect of vmPFC stimulation may have yielded an increased remote activation of the distally interconnected ventral midbrain, which in turn could disinhibit subcortical dopamine release as postulated by^49^. Both increase of GABA and decrease of Glx remote activation most likely manifested behaviorally as changes in participants’ emotional ratings. However, we report that stimulation makes perceived happiness more sensitive to changes in Glx. The increase of happiness in the active group across sessions suggests that tDCS anodal stimulation over the vmPFC and cathodal over DLPFC modulates the experience of this emotion. This interpretation is in line with the notion that vmPFC—which includes the Medial Orbitofrontal Cortex (MOFC)^54^—is crucial for emotional experience in social contexts. In fact, the MOFC plays a pivotal role in emotion in relation to reward values by integrating sensory and abstract aspects of stimuli into behavioral goals^55^. The MOFC also encodes emotional stimuli^56^.

Due to the size of the electrodes and the bipolar scalp electrode organization, there are intrinsic spatial uncertainties in measuring the effects of tDCS on emotions and behavior^57^. In this study tDCS stimulation evoked changes in reported happiness of the active group compared to sham, but did not find selective effects on guilt. It is well-established that tDCS does not only affect the brain regions directly under the electrodes but may also modulate connectivity among remote and functionally associated brain areas^58^ by influencing the strength of network connectivity^60^. Furthermore, since the MPFC is associated with representing other’s beliefs and emotions and related to social cognition processes^61^, the donation task could have induced the rise of self-perception of happiness.

We reported a non-selective decrease in guiltiness over time (sessions) in both the active and the sham groups. Feelings of guilt are associated with activation of the MPFC, among other brain regions^61^. Furthermore, the Frontopolar Cortex (BA 10)—which is part of vmPFC^28^—has been consistently found to be involved in moral judgments^62,63^and prosocial sentiments such as guilt^64–66^. In our experiment anodal tDCS over vmPFC did not influence the feeling of guilt, however. Instead, we found a decrease of guiltiness over time in both active and sham groups. This non-specific effect could be the result of other factors, for example, the act of altruistic donation on itself during the course of a week.

One caveat worth mentioning is that while all participants were informed that they would be stimulated, they were not asked about their levels of awareness of the group they were assigned. The second caveat is that as a single-blind, sham-controlled study; the participants were all told they would be receiving active stimulation, but the experimenters were aware of the actual group allocation (active/sham). The third limitation is that long MRI acquisitions had an effect on our data, as movements could negatively affect the MRS signal. This is especially critical for MRS obtained from cortical areas next to brain-bone-air interfaces, as was the case in our study. Strict analysis of signal distortion led to the loss of almost 50% of the spectroscopy data, which further reduced the power of our study in regard to the MRS findings. Considering that the neuroanatomy and scalp electrode impedance (tissue resistance using electrodes) of each participant is different, the fourth caveat is the difficulty in controlling the current flow in each specific stimulated region affected by the tDCS electrodes.

Furthermore, the effects on Glx should be interpreted with caution, as the Glx complex describes the contributions of two metabolites, i.e. glutamate and glutamine wherein the glutamate concentration in the brain is up to 45% higher than the glutamine concentration^67^. Moreover, glutamate is not only the primary excitatory neurotransmitter in the brain, but it is also implicated in the amino acid synthesis of GABA^68,69^. Thus, given that the MRS Glx signal contains contributions from several glutamate pools, it was not possible to separate the spectral contributions of glutamate proper from those resulting from the other glutamate pools.

Decision-making tasks—similar to our donation task—that require higher-level reasoning often recruit DLPFC^70^ which was under our cathodal electrode. With this in mind, future work must take into account how such emotion-laden decision tasks might interact with electrode placement and polarity. In conclusion, we provide a test-case of how a network of interconnected prefrontal brain areas can be stimulated with tDCS to influence prosocial emotional responses associated with altruistic decisions. Our findings imply that anodal stimulation of vmPFC and cathodal stimulation of right DLPFC can be used to induce remote changes in the reward network through GABA and Glx interactions with other metabolites in regions deep within the brain, which were conventionally thought to be hard to modify with tDCS.

## Methods

### Participants

Forty participants (20 women) living in Rio de Janeiro, Brazil (mean age = 24.4 ± 10; range = 19–34 years old) were recruited. The constraints of the neurostimulation and MRI protocols, a personal history of epilepsy, a cardiac pacemaker, previous intracranial surgery, pregnancy, regular psychotropics intake or inability to give informed consent were exclusion criteria. Due to the complexity of the tasks, educational level was used as an inclusion criterion: participants were undergraduate students or held a university degree. The study was approved under ethics protocol number 2.036.768 at D’Or Institute for Research and Education, Rio de Janeiro, Brazil, where the research was conducted. All experiments were performed in accordance with relevant guidelines and regulations. Informed consent was obtained from all participants.

### Protocol overview

This was a single-blind, sham-controlled study. A between-subjects design was employed, in which participants took part in a donation task on 5 consecutive days (sessions). The active group received 2 mA tDCS stimulation for 20 min (comprising 30 s ramp-up + 19 min of 2 mA stimulation + 30 s ramp-down) from the start of the session while performing a donation task on the computer. For the sham group the same stimulation lasted 1 min (comprising 30 s ramp-up and 30 s ramp-down). MRS, resting state functional connectivity (rs-fcMRI) and Diffusion Weighted Imaging (DWI) were performed on day 1—before any stimulation session—and day 5—after the last session. rs-fcMRI and DWI results will be reported elsewhere. Experimental groups were pseudo-randomly created as follows. The first participant received an identification number and was randomly allocated to one of the experimental groups, the next participant was allocated to the other group and so on, always pairing the sex ratio in both groups (Fig. 1).

### MRS acquisition

**Figure 4:**
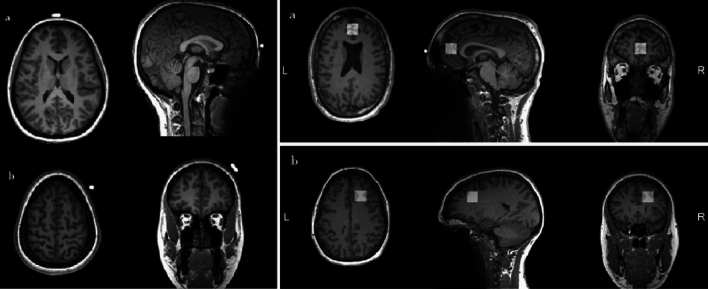
Example of MRS voxel positioning in a participant on (**a**) vmPFC, medial BA 10 and (**b**) right DLPFC, lateral BA 9.

MR images and spectra were acquired on a 3 T PRISMA scanner (Siemens Healthcare, Erlangen), using a 64-channel receive-only head coil. After the recording of a scout image, high-resolution anatomical images were acquired using a three-dimensional *T*_1_-weighted magnetization-prepared rapid acquisition gradient echo (MPRAGE) sequence (repetition time (TR), 1800 ms; echo time (TE), 2,26 ms; inversion time, 900 ms; flip angle, 8 deg; 256 × 256 matrix; 1 mm^3^ isotropic voxel; 176 slices in sagittal orientation with no gap; FOV 256 mm). MRS images were acquired on session 1 before the tDCS and donation task and on session 5 after the tDCS and the task. MPRAGE T1 was used to place the voxel of interest (20 × 20 × 20 mm^3^) over the corresponding areas of the vmPFC and the DLPFC^71,73^ based on capsules placed over the two corresponding regions of the scalp (Fig. 4). The medial voxel was positioned in the parenchyma in front of the genu of the corpus callosum, aligned with the vmPFC, medial BA 10. The other voxel was placed in the region comprehending the right DLPFC, lateral BA 9. For MRS, first the transmitter radio frequency voltage was calibrated for the individual volume of interest, followed by the adjustment of all first- and second-order shims using FAST(EST)MAP^72,73^. The consistency of voxel placement was checked between sessions with a localizer check and, if necessary, the fastmap was updated. The vmPFC voxel was acquired first, and the DLPFC voxel was acquired subsequently. GABA-edited spectra were recorded using the MEGA-PRESS technique^74^ (TR 2000 ms; TE 68,00 ms; Averages 80; Excite flip angle 90 deg; Refocus flip angle 180 deg). The water suppression was performed with VAPOR^75^.

### Transcranial direct current stimulation (tDCS)

**Figure 5:**
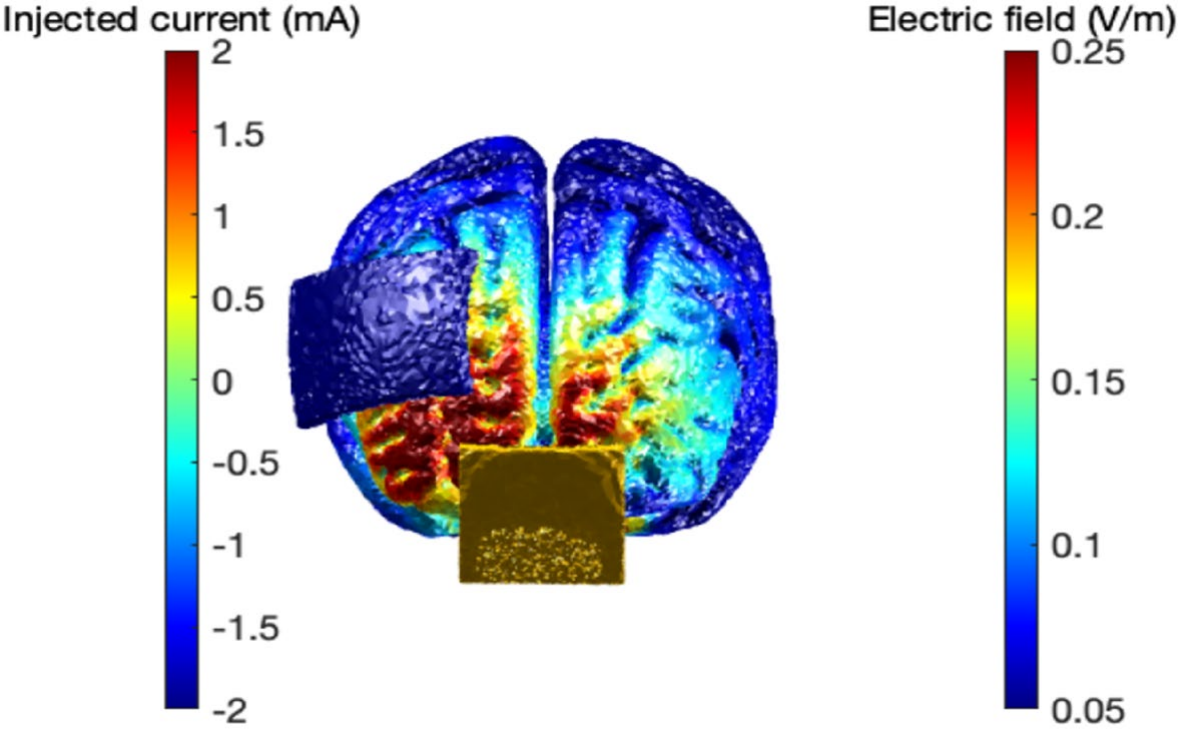
Model of the magnitude of the total electric field due to stimulation was made with ROAST^79^.

Participants were either stimulated with tDCS (active group) or received sham stimulation (sham group), while they took part in a donation task (details below). DC-STIMULATOR PLUS (NeuroConn GmbH) electrodes were placed on the corresponding area BA 10 (anode) and right lateral BA 9, which is part of the dorsolateral prefrontal cortex (rDLPFC; cathode, Fig. 5); the electrodes measured 20 cm^2^ and 15 cm^2^, the sponges were wet with saline solution and current intensity was 2mA^49^. For the vmPFC electrode placement, we used the midpoint of sites Fp1 and Fp2 of the 10/20 EEG convention, similar to studies that stimulated OFC bilaterally (e.g. ^76^). The DLPFC electrode position was defined and estimated by the program Beam F3^77^ which takes into account three head measures: circumference ear to ear, over top and inion-nasion. The electrode placement correlated with F4 site of the 10/20 EEG convention. Both groups were instructed that they would be stimulated for 20 min while they were performing a decision-making task. Concurrent with the task, the active group received stimulation of 2 mA during 20 min with a ramp up and a ramp down of 30 s, while the sham group was stimulated with a ramp up of 30 s, and a ramp down of 30 s^78^. To confirm the topographical effects of neuromodulation, we modeled the magnitude of the total electric field due to stimulation with ROAST^79^. The model provided evidence that the tDCS electric field was largest over the right vmPFC region.

The impedance was recorded for each participant, and the mean impedance of the subgroup of analysis was ~ 3,17. The electrodes position was defined and estimated by the program Beam F3^77^ which takes into account three head measures: circumference ear to ear, over top and inion-nasion.

### Donation task

**Figure 6:**
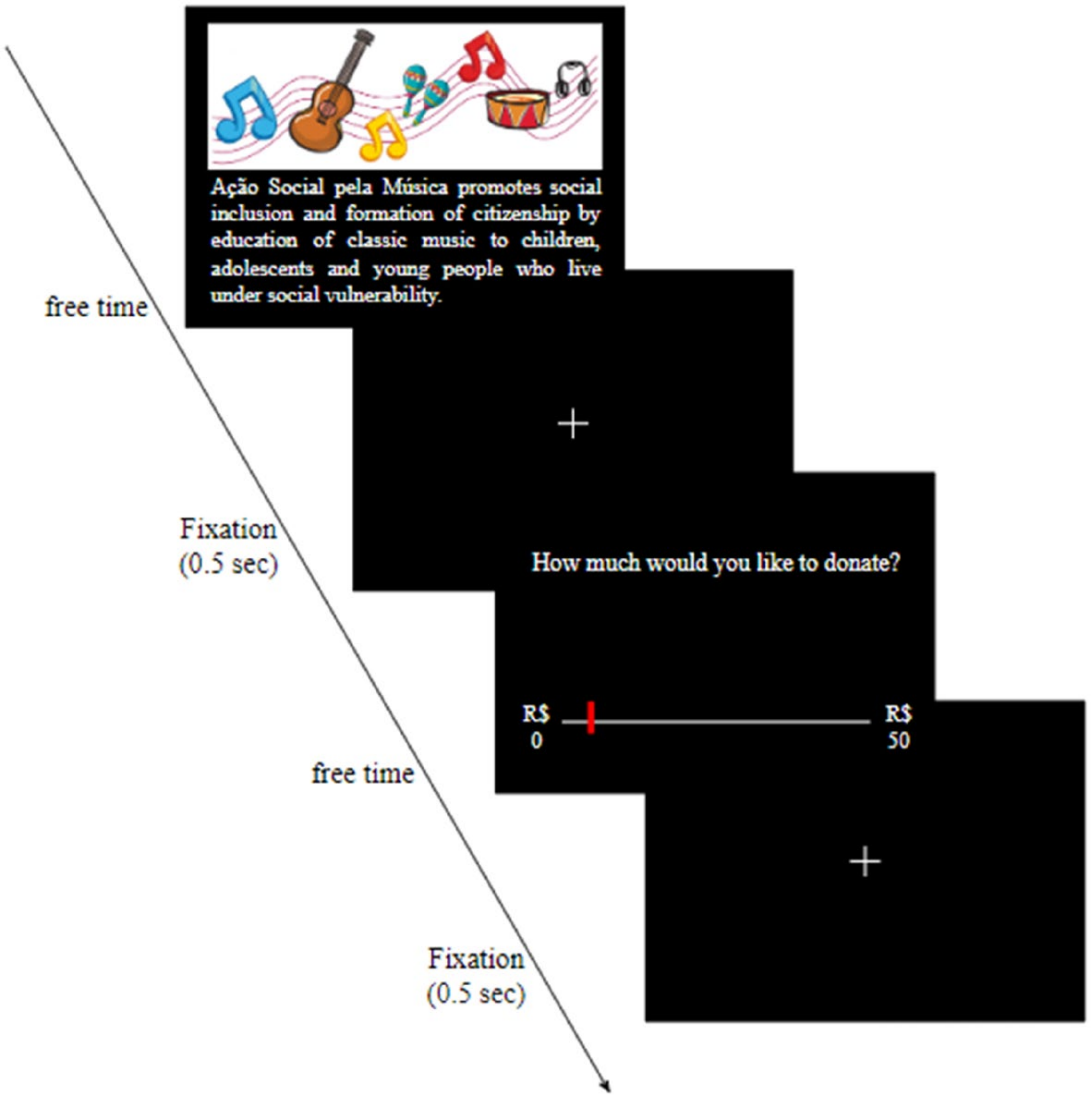
Donation task—On the first day there were 2 training trials with the same structure as the actual task. In each trial participants were presented with a photo representing the NGO, its name, and a brief description of its cause and target audience (e.g. “*Ação Social pela Música* promotes social inclusion and formation of citizenship by education of classic music to children, adolescents and young people who live under social vulnerability”. After reading the description with no time constraints, participants had to decide whether to donate any amount of money ranging from 0 to 50 Brazilian Reais for that NGO.

The task was delivered in Presentation® (Version 18.0, Neurobehavioral Systems, Inc., Berkeley, CA, www.neurobs.com). In a modified Dictator Game, 50 Brazilian Non-Governmental Organizations (NGOs) were presented per day throughout 5 days of experiment, totaling 250 NGOs. The task consisted of the participants deciding how much money they wanted to give to each NGOs. A hundred NGOs were real, while the remaining were created solely for experimental purposes. The created NGOs were described in a similar way as the real ones (Fig. 6) and participants were informed that all NGOs were real. The NGOs supported different causes: animal welfare (29.2%), humanitarian (53.6%) and “controversial” (17.2%; topics that currently have less consensus; i.e.: guns, ethnic issues, abortion, etc.). Before starting the experiment, participants read an explanation sheet about the task (Supplementary Material S1: Donation task explanation) and after confirming verbally that they understood the task, the tDCS electrodes were positioned on their heads. They were informed that they had earned R$50 and could donate any amount from R$0 to R$50 to each of the 50 NGOs presented to them during each of the five sessions. As the task was self-paced, the entire session lasted between 25 and 35 min. In addition, they were informed that one of the 250 donation trials (50 trials X 5 Sessions) would be drawn on the final day and the amount given on that trial would be donated to the respective NGO. The remaining amount would be given to the participant. For example, if the participant donated R$30 to the drawn NGO, this institution would gain $30 and the participant would gain R$20. (see Supplementary Material S1).

**Figure 7:**
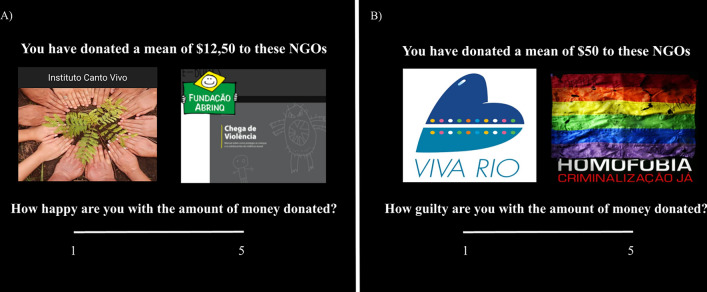
Happiness and Guiltiness ratings; at the end of the donation task, participants were asked about how happy (panel A) and guilty (panel B) they felt about the amounts of money they have donated to the various NGOs.

After each session of 50 donations, participants self-reported how happy and guilty they felt (from 1 to 5) regarding the amount of money donated to the various NGOs (Fig. 7). The NGOs were grouped in quartiles according to the average amount donated by participants. For example, the 25% of NGOs that received the least money represented the 1st quartile, while the 25% of NGOs that received the most money were grouped in the 4th quartile. That is, participants responded 4 times in each session about their feelings.

### MR spectroscopy analyses

**Figure 8:**
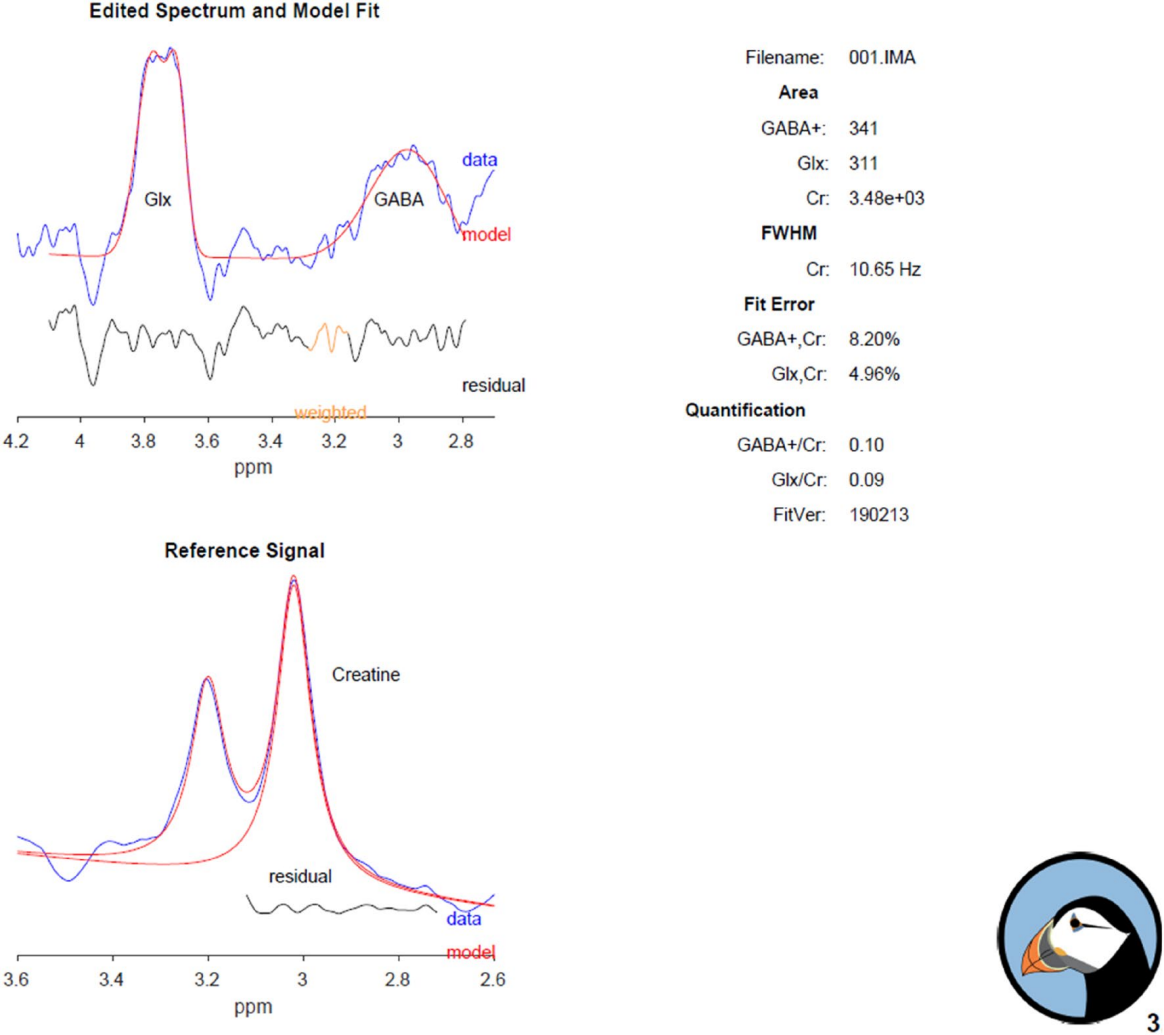
A sample magnetic resonance spectrum for GannetFit: the area under the edited GABA signal at 3 ppm and Cr signal at 3 ppm were estimated.

Edited spectra were analyzed using Gannet^80^ to estimate GABA, Glx and creatine (Cr) levels (Fig. 8); no water reference data was collected. Nonlinear least-squares fitting was used to model the difference spectrum between 2.79 and 4.10 ppm with a three-Gaussian function using a nonlinear baseline to fit the 3.0 ppm GABA and the 3.75 ppm Glx signals^81^. Quantification of GABA was estimated as the integral ratio between GABA + (GABA + co-edited macromolecules) and Cr. This ratio (hereafter “GABA”) was used as the variable of interest in the analysis described below^80^.

### Supplementary Information

Supplementary Information.

## Data Availability

The data are available upon reasonable request on the e-mail mugnol.luiza@gmail.com.
