# Supplementary material for: Transcranial electrical stimulation modulates emotional experience and metabolites in the prefrontal cortex in a donation task
Source: Sci Rep. 2024 Jun 20;14:14271. doi: 10.1038/s41598-024-64876-x (PMC11190244; doi:10.1038/s41598-024-64876-x)
Supplement: Supplementary file 1 — Supplementary Information. [file 41598_2024_64876_MOESM1_ESM.docx]

**SUPPLEMENTARY INFORMATION**

Transcranial electrical stimulation modulates emotional experience and metabolites in the prefrontal cortex in a donation task

**Supplementary Method S1: Donation Task Explanation**

The participant read the following text on the computer screen:

******

**Hello!**

Thank you very much for your participation, science thank you.

You will participate in a task where you will donate money to NGOs. In each donation, you will have R$50 available to donate. We ask that you treat each donation **as unique**.

Make each donation as if you were using your **own money**. Please use the entire donation scale (from R$0 to R$50), that is, evaluate how much each NGO **deserves**.

At the end of the experiment, you'll see the summary of your donations. At this point, we will ask you how happy or guilty you felt when making those donations. Please be aware of these two feelings and indicate your response on the scale.

On the last day of the experiment, we will randomly assign 1 NGO to which we will donate the money corresponding to your choice, and you will get the rest.

In addition, by participating, you will receive R$100

· R$12 per day (x 5) for participation = R$60

· R$8 per day (x 5) for the transportation to the institute = R$40

**

**Supplementary Method S2: Additional Analysis**

To examine the potential relationship between prefrontal metabolic changes due to the stimulation protocol and the emotions following altruistic experience, we ran four regression analyses. These tested whether changes in GABA predicted changes in reported happiness and guilt and whether there was an influence of the stimulation group. While there are significant main effects of the group, none of the main effects of change in metabolite was significant (all p’s>.204), nor was there an interaction between the two factors (all p’s>0.135) (Figure S1 A-D). We ran four additional regression analyses to test the same relation for changes in Glx. There was a significant interaction between the change in reported happiness and Glx in vmPFC. For the sham group changes in Glx did not lead to changes in reported happiness, t(16)=.358, p=.725. For the stimulation group, however, the larger the increase in Glx, the larger the increase in the reported happiness t(16)=4.562, p<.001. While there are significant main effects of group and change in metabolite in the other three regressions, none found significant interactions between these two factors (all p’s>0.554). (Figure S1 E-H).


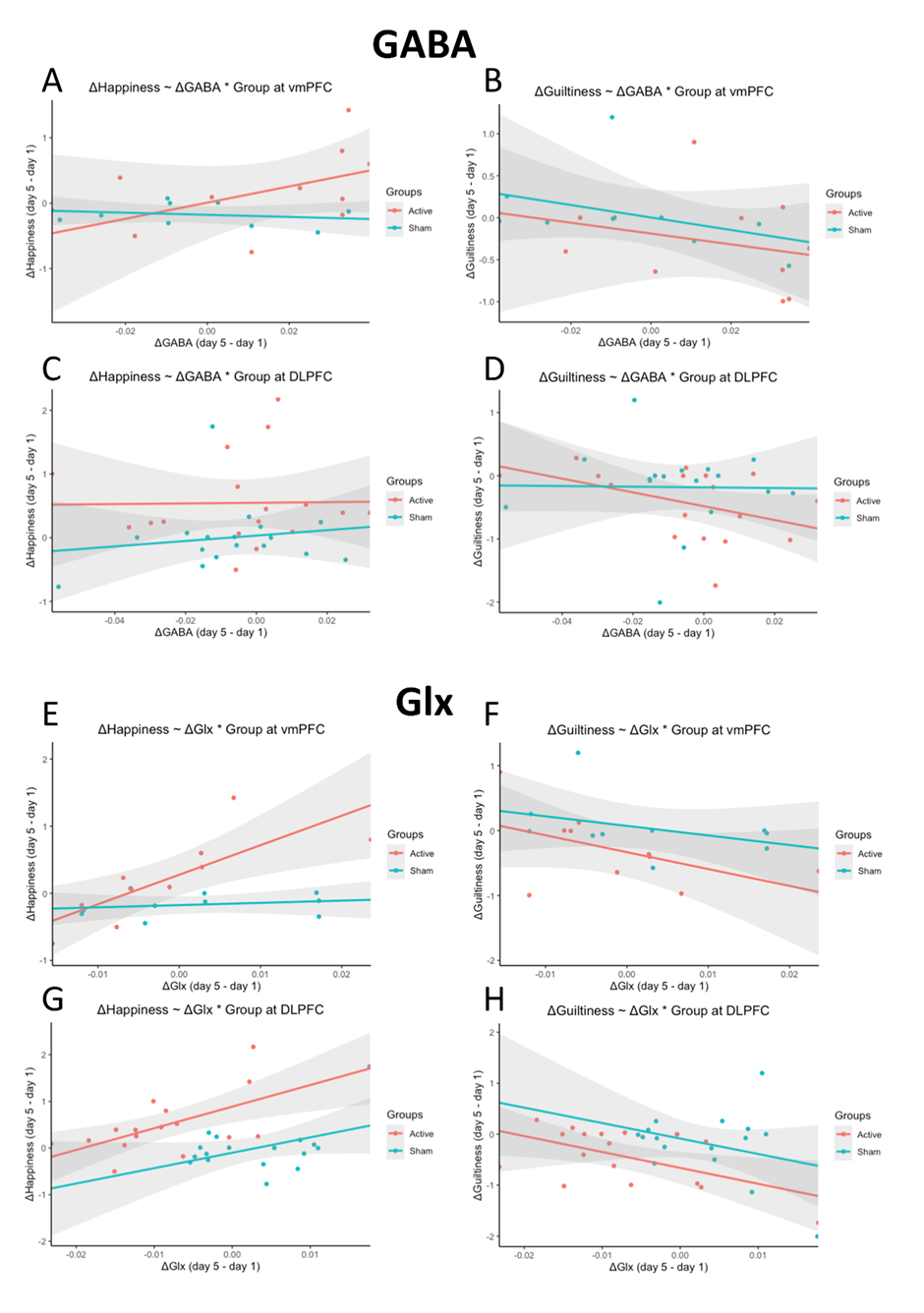


**Supplementary Figure S1:** Changes in reported happiness as a function of changes in GABA and stimulation group for (A) vmPFC; (B) DLPFC. Changes in reported guilt as a function of changes in GABA and stimulation group for (C) vmPFC; (D) DLPFC. Changes in reported happiness as a function of changes in Glx and stimulation group for (E) vmPFC; (F) DLPFC. Changes in reported guilt as a function of changes in Glx and stimulation group for (G) vmPFC; (H) DLPFC.
